# Treatment patterns and out-of-hospital healthcare resource utilisation by patients with advanced cancer living with pain: An analysis from the Stop Cancer PAIN trial

**DOI:** 10.1371/journal.pone.0282465

**Published:** 2023-02-28

**Authors:** Nikki McCaffrey, Seong Leang Cheah, Tim Luckett, Jane L. Phillips, Meera Agar, Patricia M. Davidson, Frances Boyle, Tim Shaw, David C. Currow, Melanie Lovell

**Affiliations:** 1 Deakin Health Economics, Institute for Health Transformation, Faculty of Health, Deakin University, Burwood Campus, Burwood, VIC, Australia; 2 Faculty of Health, IMPACCT (Improving Palliative, Aged and Chronic Care through Clinical Research and Translation Sydney), University of Technology Sydney (UTS), Sydney, NSW, Australia; 3 Faculty of Health, School of Nursing, Queensland University of Technology, Kelvin Grove Brisbane, Queensland; 4 Faculty of Science, Medicine and Health, University of Wollongong, Wollongong, NSW, Australia; 5 Patricia Ritchie Centre for Cancer Care and Research, Mater Hospital North Sydney, and University of Sydney, Sydney, NSW, Australia; 6 Faculty of Medicine and Health, Charles Perkins Centre, The University of Sydney, Sydney, NSW, Australia; 7 Department of Palliative Care, HammondCare, Greenwich Hospital, Sydney, NSW, Australia; 8 Northern Clinical School, University of Sydney, Sydney, NSW, Australia; Iranian Institute for Health Sciences Research, ISLAMIC REPUBLIC OF IRAN

## Abstract

**Background:**

About 70% of patients with advanced cancer experience pain. Few studies have investigated the use of healthcare in this population and the relationship between pain intensity and costs.

**Methods:**

Adults with advanced cancer and scored worst pain ≥ 2/10 on a numeric rating scale (NRS) were recruited from 6 Australian oncology/palliative care outpatient services to the Stop Cancer PAIN trial (08/15-06/19). Out-of-hospital, publicly funded services, prescriptions and costs were estimated for the three months before pain screening. Descriptive statistics summarize the clinico-demographic variables, health services and costs, treatments and pain scores. Relationships with costs were explored using Spearman correlations, Mann-Whitney U and Kruskal-Wallis tests, and a gamma log-link generalized linear model.

**Results:**

Overall, 212 participants had median worst pain scores of five (inter-quartile range 4). The most frequently prescribed medications were opioids (60.1%) and peptic ulcer/gastro-oesophageal reflux disease (GORD) drugs (51.6%). The total average healthcare cost in the three months before the census date was A$6,742 (95% CI $5,637, $7,847), approximately $27,000 annually. Men had higher mean healthcare costs than women, adjusting for age, cancer type and pain levels (men $7,872, women $4,493, p<0.01) and higher expenditure on prescriptions (men $5,559, women $2,034, p<0.01).

**Conclusions:**

In this population with pain and cancer, there was no clear relationship between healthcare costs and pain severity. These treatment patterns requiring further exploration including the prevalence of peptic ulcer/GORD drugs, and lipid lowering agents and the higher healthcare costs for men.

**Trial registration:**

ACTRN12615000064505. World Health Organisation unique trial number U1111–1164–4649. Registered 23 January 2015.

## Introduction

Approximately 70% of patients living with advanced cancer experience pain [1–3] despite the proliferation of different treatment approaches and clinical practice guidelines [4, 5]. A recent practice review of national and international guidelines describing pain management in patients with advanced cancer by Chapman and colleagues [6] suggested an oral strong opioid is preferred for treating moderate or severe pain in patients with bisphosphonates and/or radiotherapy added for bone pain. Further, the authors recommended paracetamol should not be used in conjunction with a strong opioid to treat pain, weak opioids, lidocaine and ketamine are indicated in specific situations only and non-pharmacological approaches could also play a role. The Australian guidelines are consistent with these recommendations. Commonly reported barriers to cancer pain management include negative attitudes to treatments, lack of knowledge, hesitancy to report pain, time pressures, inadequate screening processes and a lack of care coordination [7–9].

Pain is associated with poorer quality of life [10] and may increase healthcare resource utilisation and costs through increased unplanned hospital readmissions, hospitalisations, emergency department and medical attendances, and longer inpatient stays [3, 11–13]. Despite the high prevalence of pain in people living with advanced cancer, there are few contemporary, prospective studies describing healthcare resource utilisation in this population, particularly in countries with largely universal health coverage such as Australia, Canada or the United Kingdom.

Cancer is the leading cause of social and economic burden in Australia [14], with direct annual health service costs estimated at A$7 billion [15]. Whilst there is a substantial body of evidence on the broader economic burden of cancer, there is a lack of information specific to cancer pain. As demands on healthcare systems escalate [16], information on how best to invest limited resources is needed to optimise the value of care for patients, family members, clinicians and societal decision-makers [17]. Understanding current patterns in healthcare use is critical for informing resource allocation decisions and developing sustainable health policy. Healthcare utilisation research can also inform assessment of the quality of care and quality use of medicines, and identify areas of potential sub-optimal treatment [18–20]. Further, greater understanding of patterns of medical service use and prescriptions can inform policies to mitigate escalating out-of-pocket costs (patient costs due to the gap between the cost of the service and the amount reimbursed under Medicare) for people affected by cancer. This financial burden can lead to increasing stress, poorer quality of life and lower rates of access to care even in countries with universal health coverage [21–25].

However, no studies have investigated the relationship between healthcare usage and pain intensity in people with advanced cancer in Australia. Such estimates facilitate modelling of the effect of successfully reducing pain levels on subsequent healthcare utilization through improved pain management. For example, these estimates would usefully inform economic evaluations, i.e. modelled cost-effectiveness, cost-utility and cost-benefit analyses, which systematically compare the costs and benefits of competing strategies and provide information about how best to improve outcomes within funding constraints [16, 23, 26]. Estimating the effect of successfully reducing pain levels on subsequent healthcare utilization provides vital data to inform the cost inputs into these types of modelled evaluations and provide decision makers with vital knowledge to inform future service delivery.

Additionally, there is a paucity of research on factors associated with healthcare utilisation and costs in patients living with advanced cancer pain in Australia such as patient, disease or pain-related characteristics. This knowledge could help identify potential opportunities for improvement in cancer pain management. For example, data from the United States (US) suggests that younger age, lower income level and greater pain intensity are associated with higher healthcare costs in outpatients experiencing cancer-related pain [11], and that age, sex, stage of cancer, comorbidities, year of diagnosis and income predict hospital length of stay for people with cancer admitted to hospital for pain management (direction of effect not reported by Alese and colleagues; abstract only) [27]. However, these findings may not be generalisable to other countries such as Australia given the different ways healthcare is funded, most notably the difference between predominantly private insurance in the US and publicly financed health insurance (Medicare) in Australia [28]. As healthcare is highly contextual to settings, understanding treatment patterns, i.e. use of types of health service and medications, is important in distinct jurisdictions.

### Aim

The aims of this study were to:

1. identify treatment patterns and corresponding costs of healthcare resource use (government funded) for outpatients living with advanced cancer and pain;
2. explore factors associated with healthcare costs in this population; and
3. examine the relationship between healthcare costs and pain intensity.

## Methods

### The Stop Cancer PAIN Trial

This pragmatic, phase III, stepped-wedge, cluster randomised controlled trial investigated the effectiveness of screening and guidelines for pain with, versus without, implementation strategies for improving cancer pain.

From August 2015 to June 2019, adults with cancer and pain presenting to six oncology and palliative care outpatient services across Australia were recruited to the Stop Cancer PAIN Trial [8]. Each cluster (i.e., participating oncology and/ or palliative care outpatient service (n = 6)), was randomized to introduce the implementation strategies (i.e., audit and feedback, clinician-spaced education, and a patient self-management resource), at different times following an initial control period [9]. Recruitment and measurement were placed on hold during a training phase transition from control to intervention. The complete details of the trial are published elsewhere [8].

To be eligible, patients had to be, outpatients with a diagnosis of advanced cancer, the ability to self-complete the 0–10 numeric rating scale (NRS) for severity of worst and average pain, reporting a worst pain score of ≥5 (primary outcome) or ≥2 (secondary outcome) and sufficient proficiency in spoken English to complete the secondary outcome measures were eligible to participate in the study [8].

De-identified pain screening data from *all* patients attending services during the study period was permitted to avoid selection bias [1]. The Stop Cancer PAIN Trial applied an opt-out procedure for patients’ permission to obtain their contact details and telephone them one week later for verbal informed consent to provide primary and secondary outcome data. Consequently, there were two patient participant populations in the study: the first contributed to the primary outcome data; and the second to the secondary outcomes.

The primary outcome was the proportion of participants initially reporting a worst pain score of ≥5 who experienced a clinically important improvement of ≥30% 1 week later. Secondary outcomes included mean average pain, quality of life, patient empowerment, and fidelity to the intervention, and were measured in all participants initially reporting a worst pain score of ≥2 at weeks one, two, and four. Overall, there was no statistical difference in pain-related outcomes; the implementation strategies were insufficient to improve pain-related outcomes for outpatients with cancer-related pain [9].

### Treatment patterns and out-of-hospital healthcare resource utilisation

The Stop Cancer PAIN Trial database was linked to routinely collected out of hospital services (Medicare Benefits Schedule) and medication data (Pharmaceutical Benefits Schedule) to explore treatment patterns and estimate healthcare resource utilisation. Ethics approval was granted by the South Western Sydney Local Health District Human Research Ethics Committee (HREC)–ethics approval number HREC/14/LPOOL/479 and the data custodian, the Australian Department of Human Services External Request Evaluation Committee (MI4457). Study participants provided written informed consent for access to these data.

Approval was granted by the HREC for an opt-out procedure to contact patients at week one as well as a procedure to obtain informed verbal (rather than written) consent to participate. Verbal consent was considered to place less burden on patients who, due to their illness, might be less able to return written consent forms by mail.

### Medicare Benefits Schedule (MBS) and Pharmaceutical Benefits Schedule (PBS)

Medicare is the publicly funded universal healthcare scheme in Australia providing access to subsidised medical services for all residents [29]. Approved, subsidised services such as attendances by medical doctors and allied health professional, and investigations are listed in the Medicare Benefits Schedule (MBS). Services are typically privately provided on a fee-for-service basis. The MBS describes the type of service provided and the amount reimbursed by the government [30]. The MBS fee, set by the Australian Government, may differ from the provider’s actual fee and, if so, the patient pays any difference between the two (“out-of-pocket” cost), up to an annually defined “safety net” (A$477.90 in 2020). If the Medicare benefit is accepted by the provider as full payment, there is no out-of-pocket cost (termed ‘bulk-billed’). The Australian Government also subsidises approved medications which are listed in the Pharmaceutical Benefits Schedule (PBS) with an annual safety net of A$316.80. Generally, patients contribute a co-payment [29], A$41 for general patients and A$6.60 for concessional patients (December 2020), and the government funds the remainder of the cost [31]. The Australian Government Department of Human Services maintains a database which captures MBS and PBS usage information, including the type of service, drug classification, amount reimbursed and date of supply.

### Outcome measures

Participants demographics and cancer diagnoses details were collected [8].

#### Pain Numeric Rating Scale (NRS)

The pain NRS is a widely used, self-completed, validated pain scale [32, 33]. The single-item scale has 11 categories ranging from 0 (no pain) to 10 (worst pain you can imagine). Ratings of 7–10 are considered to represent severe pain, 5–6 moderate pain, and 1–4 mild pain, according to the corresponding impact on functioning [8, 34, 35]. Study participants completed the pain NRS for worst and average pain over the last 24 hours at baseline, weeks one, two and four.

#### EORTC QLQ C15-PAL

The European Organisation for Research and Treatment of Cancer Quality of Life-C15-Palliative questionnaire (‘C15-PAL’) is a shortened version of the QLQ-C30, and contains 15 of the 30 original items [36]. The C15-PAL questionnaire has 14 items (each with four possible responses, not at all = 1, a little = 2, quite a bit = 3, and very much = 4) which are grouped into two functional scales (physical and emotional), five single-item symptom scales (dyspnoea, insomnia, appetite loss, constipation, nausea and vomiting), two multi-item symptom scales (pain and fatigue) and an overall quality of life rating scale with seven categories ranging from 0 (very poor) to 7 (excellent) [36]. A scoring algorithm is used to convert the response categories to a score (0–100), where lower scores indicate a reduced symptom burden, reduced levels of functioning and lower HrQOL [37, 38]. The C15-PAL is a reliable, responsive and valid measure in advanced cancer settings [39–48] but does not produce an overall total score derived from all the items. Responses to the C15-PAL questionnaire were collected at weeks one, two and four for participants who consented to provide secondary outcome data [49, 50]. Due to unacceptable participant and research staff burden and consent procedures, C15-PAL responses were not collected at baseline.

#### EORTC QLU-C10D

The C15-PAL does not provide a total score reflecting the health-related quality of life of people living with cancer and cannot be used to inform economic evaluations because this patient-reported outcome measure is not preference based, i.e. does not enable the calculation of utility values. The EORTC QLU-C10D [51] (referred to as ‘QLU-C10D’ hence) *is* a preference-based instrument developed from the widely used cancer-specific quality-of-life (QOL) questionnaire, EORTC QLQ-C30 [50] and enables estimation of utilities to inform economic evaluation. Thirteen of the 30 EORTC QLQ-C30 items are combined into ten dimensions, each with four levels: physical, role, social, and emotional functioning; pain; fatigue; sleep; appetite; nausea; and bowel problems [51]. As the Stop Cancer PAIN Trial participants had advanced cancer, the C15-PAL rather than the QLQ-C30 was used to measure HrQOL. The C15-PAL includes eight of the 13 required items for calculating QLU-C10D utility scores [51]. Consequently, responses to five additional items were also collected to enable estimation of QLU-C10D utilities. The Australian scoring algorithm [52] was used for calculating utility weights.

### Data linkage

Medicare Benefit Schedule and PBS data were requested for all consented participants. The Department of Human Services carried out probabilistic linkage to the Stop Cancer PAIN Trial ID with the MBS and PBS database based on key variables such as date of birth and Medicare number and provided anonymised data to the lead investigator. Data were extracted on 27 November 2019. Services provided through public hospitals such as inpatient, outpatient or emergency care were not recorded in the Stop Cancer PAIN Trial and therefore are not included in the analyses. Consistent with previous Australian healthcare resource utilisation studies, services provided to Department of Veterans Affairs beneficiaries were also excluded because of greater range of services accessible to these beneficiaries compared with the average Australian [15, 53–55].

### Analysis

Analyses were performed using SPSS for Windows version 25.0 (SPSS, Inc., Chicago, IL) and Stata version 16.0 (StataCorp. 2019. Stata Statistical Software: Release 16. College Station, TX: StataCorp LLC) on available data. Descriptive summary statistics were estimated for clinico-demographic variables, NRS pain and HrQOL scores, QLU-C10D utilities and healthcare resource use and costs.

Medical and allied health services were categorised consistent with the Medicare Benefits Schedule (See Table 1 in S1 Appendix for categories) [30]. Medications were categorised according to the World Health Organisation Anatomical Therapeutic Chemical (ATC) classification system [56] which categorises medications according to their pharmacological, chemical and therapeutic properties and the system or organ on which they impact. The total cost of medical care for each participant was estimated from the Medicare benefits paid for the three months prior to the screening date commensurate with previous cost analyses [11, 57–59]. Similarly, the total cost of medications was estimated from the net benefits paid for the same time period, i.e., the actual cost of the medicine, also known as the “dispensed price for maximum quantity” (DPMQ) less the patient co-payment. For example, the DPMQ for a pack of imatinib 400mg tablets [30] was A$946.71 in 2020, a general patient co-payment was A$41 and therefore the net benefit paid by the government was A$905.71. The supply dates for medical services and medications were used to determine the relevant time period as this is the essentially the date of purchase. Average medical service utilisation was compared with the Australian population using customised Medicare and Pharmaceutical Benefits Scheme statistics reports produced by Services Australia (freely available from http://medicarestatistics.humanservices.gov.au/statistics/mbs_group.jsp).

Healthcare costs were positively skewed. Consequently, *a priori* hypothesised correlations (presented in brackets), based on the available literature and clinical expertise, between total healthcare costs and age (weak, positive), pain intensity (moderate, positive), HrQoL scores (weak, negative) and QLU-C10D scores (weak, negative) [11, 60, 61] were evaluated with Spearman’s correlation coefficients (>0.50 strong; 0.30–0.50 moderate; <0.30 weak [62]).

Differences between clinico-demographic sub-groups were assessed using the non-parametric Mann Whitney *U* test (two groups) and Kruskal-Wallis one way analysis of variance (multiple groups). Healthcare costs were expected *a priori* to be associated with gender and cancer type [61, 63].

The relationships between clinico-demographic variables and healthcare resource utilisation were further explored using a generalised linear model (GLM) with a gamma distribution and a log link [64]. This model controls for skewness, non-negative values and approximates the distribution of data according to the modified Park test [65]. Age and gender were entered first in the model (Model 1). Subsequently, an exploratory analysis was conducted to assess whether age, sex, cancer type and pain category were independently associated with healthcare costs (Model 2). Model goodness-of-fit was evaluated using the Akaike information criterion (AIC) and Bayesian information criterion (BIC) where smaller values suggest a better-fitting model [66]. As interaction terms were not statistically significant in simple, exploratory linear regression analyses, no interaction terms were included in the GLM [67]. The significance level for all analyses was set a *p*<0.05 with two-sided significance tests.

## Results

### Sample characteristics

In total, 30.8% (n = 212) of patients who participated in the Stop Cancer PAIN Trial consented to having their MBS and/ or PBS data accessed. Overall, total healthcare costs and Stop Cancer PAIN Trial data were available for 186 participants (MBS costs, n = 186, PBS costs n = 188) for this study (missing trial data, n = 26). All costs are reported as Australian dollars.

The participant demographics and clinical characteristics are summarised in Table 1; the mean age was 64.5 years, 45.3% were female and the most prevalent cancer types were genitourinary (21.5%), breast (17.5%) and gastrointestinal (16.4%). Participants who consented to having their MBS and/ or PBS data accessed were generally similar to the Stop Cancer PAIN Trial sample except more men (51.0% vs 54.7%) and a higher proportion of participants diagnosed with a genitourinary cancer (17.9% vs 21.5%) consented to access to MBS and/ or PBS data.

**Table 1:** Participant demographics and clinical characteristics.

	Both groups N = 212	Control N = 125	Intervention N = 87
Age, years (mean, SD)	64.5 (10.7) n = 210	65.5 (9.7) n = 124	63.0 (11.8) n = 86
Sex, females (n, %)	96 (45.3)	52 (41.6)	44 (50.6)
Cancer Type (n, %)			
Breast	37 (17.5)	20 (16.5)	17 (19.5)
Lung	31 (14.5)	22 (17.6)	9 (10.3)
Head and neck	15 (7.0)	9 (7.2)	6 (6.9)
Other	33 (15.4)	15 (12.0)	17 (19.5)
Gastrointestinal	35 (16.4)	21 (16.8)	14 (16.1)
Genotiurinary	46 (21.5)	28 (22.4)	18 (20.7)
Haematologic	8 (3.7)	5 (4.0)	3 (3.4)
Missing	8 (3.7)	5 (4.0)	3 (3.5)
Baseline pain NRS (median, IQR)	5.0 (4)	5.0 (4)	5.0 (4)

IQR = inter-quartile range; NRS = pain numerical rating scale; SD = standard deviation

#### 1. Treatment patterns and corresponding costs of healthcare resource use

**Fig 1:**
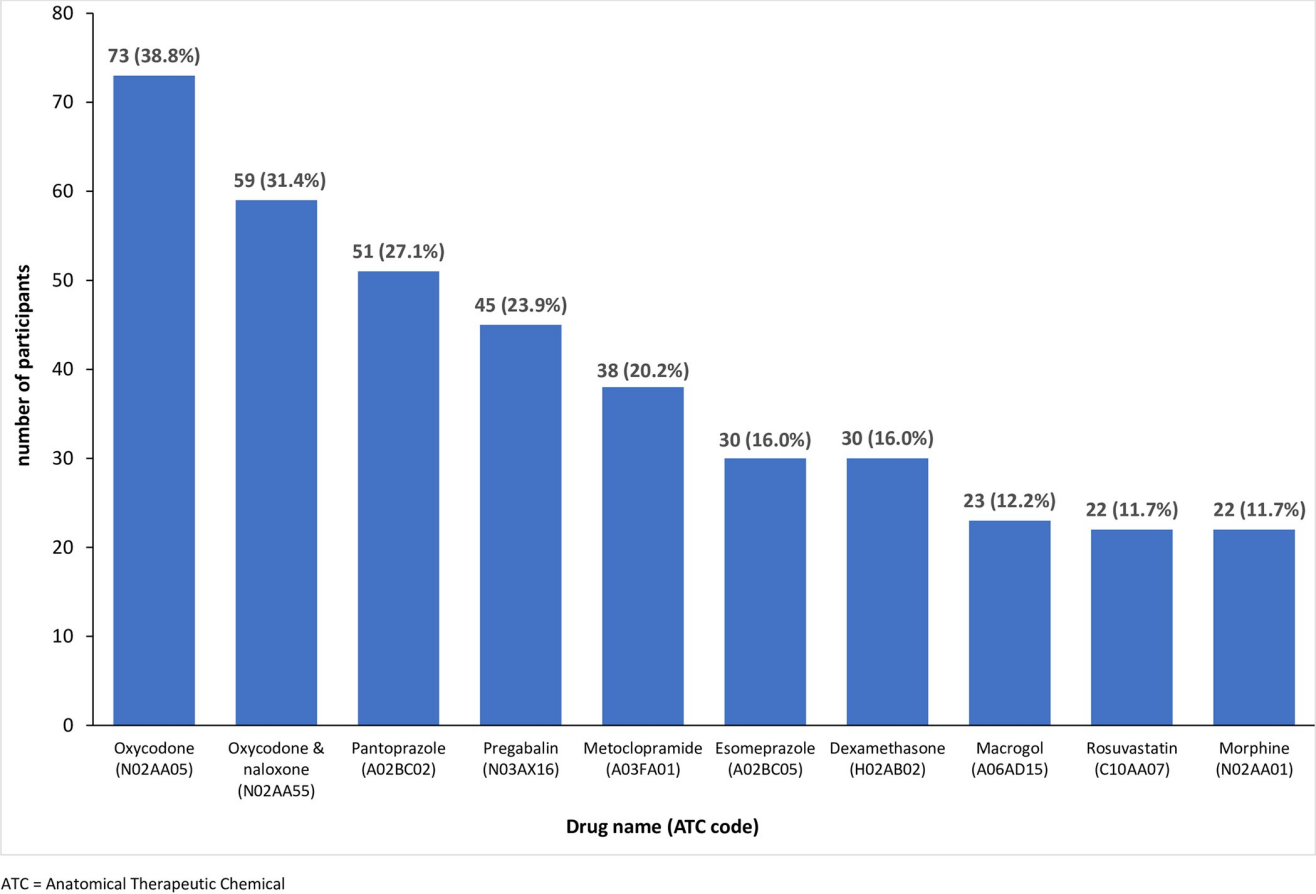
Top 10 most frequently prescribed medicines.

*Medical and allied health service use and diagnostics and pathology*. The most commonly accessed services by participants were: professional (99.5%) services such as physician attendances, case conferences, medication reviews and heath assessments; pathology (95.2%); and diagnostic imaging services (84.9%, see Fig 1 in S2 Appendix). The most commonly used services were pathology (5.6 per month), professional services (3 per month), and therapeutic procedures (2.1 per month; Table 2).

**Table 2:** Medicare benefits schedule service use and costs in the three months prior to screening.

MBS Service (N = 186)	Total no. of services	Average no. of services/ participant	Average no. of services/ participant/ month	Average no. of services/ capita/ month^#^	Total cost	Mean cost/ participant	SD	Mean cost/ participant/ month
1. Professional attendances	1696	9.1	3.0	0.6	$110,743	$595	$379	$20
2. Diagnostic procedures	68	0.4	0.1	0.0	$4,471	$24	$73	$0.80
3. Therapeutic procedures	1183	6.4	2.1	0.1	$187,862	$1,010	$1,951	$34
4. Oral & maxillofacial services	1	0.0	0.0	0.0	$73	$0.39	$5	$0.01
5. Diagnostic imaging services	68	0.4	0.1	0.1	$165,955	$892	$745	$30
6. Pathology services	3100	16.7	5.6	0.5	$54,136	$291	$267	$10
8. Miscellaneous services	70	0.4	0.1	0.1	$4,104	$22	$63	$0.74

Note, there were no services reported for “7. Cleft lip & cleft palate services”; MBS = Medicare Benefits Schedule; SD = standard deviation; ^#^ calculated using Medicare Items Report for time period Aug 2015 to Jun 2019 http://medicarestatistics.humanservices.gov.au/statistics/mbs_item.jsp

On average, participants had more professional attendances than the Australian population (3 vs 0.6 per month), more therapeutic procedures (2.1 vs 0.1 per month) and utilised a greater number of pathology services (5.6 vs 0.5 per month).

The total cost for publicly funded medical service use in the three months prior to the study was $527,345. The total average cost per participant was $2,836 (95% CI $2,489, $3,184), i.e., $945 per month. The mean cost per participant was highest for therapeutic procedures ($1,010, 95% CI $732, $1,299)), followed by diagnostic imaging ($892, 95% CI $784, $1,000) and professional attendances ($595, 95% CI $541, $650).

*Medication use*. Overall, 188 participants were supplied 3,188 prescriptions in the three months prior to study; just over two-thirds of prescriptions were concessional (65.8%), i.e. pensioners or those who had surpassed the PBS safety net, compared with 91.3% of all PBS prescriptions in 2018–19 [68] and 34.2% were general, i.e. patients who contributed the full co-payment, versus 8.5% of all PBS prescriptions.

On average, participants received more prescriptions per month than the Australian population (5.7 vs 0.3). Opioids were the most commonly supplied category of medication (60.1%), followed by drugs for peptic ulcer and gastro-oesophageal disease (51.6%) and antiepileptics (26.6%, see Fig 2 in S3 Appendix). In contrast, during 2016–2017, 3.1 million people were dispensed opioid medications in Australia, out of a population of 24,590,334 (12.6%) [69]. Further, the top three medication groups prescribed in the Australian general population were agents acting on the renin-angiotensin system (11.2%), lipid modifying agents (10.5%) and psychoanaleptics (8.3%), and 7.8% of prescriptions were for analgesics. Oxycodone (38.8%), oxycodone and naloxone (31.4%) and pantoprazole (27.1%) were the most common medications received by participants (Table 3), whereas rosuvastatin (3.7%), atorvastatin (3.6%) and pantoprazole (2.7%) were the top three medications prescribed in the Australian general population.

**Table 3:** Types and costs of medications prescribed in the three months prior to screening.

	Total number of prescriptions	Average no of prescriptions/ participant	Average no of prescriptions/ participant/ month	Total cost	Mean cost/ participant	SD	Mean cost/ participant/ month
**PBS (N = 188)**	3188	17.0	5.7	$731,327.66	$3,890.04	$7,149.63	$1,296.68
**Anatomical level/ therapeutic area (ATC code)**							
Opioids (N02A)	585	3.1	0.5	$18,499.40	$98.40	$168.60	$32.80
Drugs for peptic ulcer & GORD (A02B)	255	1.4	0.1	$2,549.53	$13.56	$25.97	$4.52
Antiepileptics (N03A)	128	0.7	0.2	$3,173.63	$16.88	$44.96	$5.63
Corticosteroids for systemic use (H02A)	99	0.5	0.2	$608.06	$3.23	$9.75	$1.08
Other antineoplastic agents (L01X)	178	0.9	0.2	$384,332.32	$2,044.32	$6,492.24	$681.44
Lipid modifying agents (C10A)	110	0.6	0.2	$1,487.22	$7.91	$25.99	$2.64
Propulsives (A03F)	73	0.4	0.1	$390.44	$2.08	$10.46	$0.69
Antidepressants (N06A)	118	0.6	0.3	$1,082.69	$5.76	$18.03	$1.92
Antiemetics & antinauseants (A04A)	110	0.6	1.0	$3,585.10	$19.07	$69.10	$6.36
Antithrombotic agents (B01A)	91	0.5	0.2	$8,626.64	$45.89	$138.16	$15.30
Beta-lactam antibacterials, penicillins (J01C)	41	0.2	0.2	$235.82	$1.25	$4.24	$0.42
**Top 10 most frequently prescribed medications**							
Oxycodone (N02AA05)	206	1.1	0.4	$5,112.09	$27.19	$78.54	$1.26
Oxycodone & naloxone (N02AA55)	181	1.0	0.3	$7,382.69	$39.26	$91.78	$2.17
Pantoprazole (A02BC02)	119	0.6	0.2	$710.38	$3.78	$11.51	$0.49
Pregabalin (N03AX16)	117	0.6	0.2	$2,851.49	$15.17	$42.36	$1.30
Metoclopramide (A03FA01)	62	0.3	0.1	$274.90	$1.46	$6.94	$1.05
Esomeprazole (A02BC05)	72	0.4	0.1	$1,223	$6.50	$20.28	$0.45
Dexamethasone (H02AB02)	43	0.2	0.1	$255.13	$1.36	$5.35	$4.20
Macrogol (A06AD15)	45	0.2	0.1	$733.26	$3.90	$18.69	$9.06
Rosuvastatin (C10AA07)	47	0.3	0.1	$590.86	$3.14	$11.68	$13.09
Morphine (N02AA01)	87	0.5	0.2	$2,370.94	$12.61	$54.95	$5.06

ATC = Anatomical Therapeutic Chemical; GORD = gastro-oesophageal reflux disease; PBS = Pharmaceutical Benefits Scheme; SD = standard deviation

The total cost for prescriptions was $731,327. The total average cost per participant was $3,890 (95% CI $2,861, $4,919), i.e. $1,297 per month. The mean cost per participant was highest for other antineoplastic agents ($2,044, 95% CI $1,110, $2,978), followed by opioids ($98, 95% CI $74, $122) and antithrombotic agents ($46, 95% CI $26, $66) (see Table 3).

*Total healthcare cost*. The total healthcare cost was in the three months prior to the study was $1,253,187. The total average cost per participant was $6,742 (95% CI $5,637, $7,847) or $2,247 per month.

#### 2. Factors associated with healthcare costs

Spearman’s rank correlations between total healthcare costs and age, pain intensity and HrQOL and QLU-C10D scores were in the anticipated directions but weaker than expected especially for pain intensity (Table 4). There were no statistically significant correlations except for total PBS costs and age (rho = 0.159, p = 0.03) indicating medication costs increase with age.

**Table 4:** Correlations between total healthcare, MBS and PBS costs and age, pain intensity and health-related quality of life.

Variable	Total healthcare costs (N = 186)	p-value	MBS costs (N = 186)	p-value	PBS costs (N = 188)	p-value
Age	0.021	0.778	-0.097	0.190	0.159	**0.031**
	n = 183		N = 183		N = 184	
Baseline pain NRS	0.042	0.567	0.072	0.329	0.083	0.262
	n = 185		N = 185		N = 186	
HrQOL	-0.033	0.785	0.065	0.593	-0.014	0.907
	N = 69		N = 69		N = 69	
EORTC-QLU C10D	-0.064	0.603	0.026	0.830	-0.108	0.377
	N = 69		N = 69		N = 69	

Sample sizes vary due to missing data; HrQOL = health-related quality of life; MBS = Medicare Benefits Schedule; NRS = pain numerical rating scale; PBS = Pharmaceutical Benefits Schedule; Correlations were interpreted according to Cohen’s guidelines, i.e., “strong” (≥0.51), “moderate” (0.31–0.50), “weak” (0.11–0.30), and “none” (0–0.10). Statistically significant correlations are bolded. + indicates positive direction; -, negative direction

In the bivariate analyses, there was a statistically significant difference in mean total healthcare costs for gender but not age, cancer type or baseline pain level. Mean total healthcare costs were higher for men ($7,924, 95% CI $6,267, $9,581) than women ($5,367, 95% CI $3,975, $6,760) (U = 3546, *p* = 0.04) (Table 5).

**Table 5:** Unadjusted, mean healthcare costs by clinico-demographic characteristics.

	Total			MBS			PBS		
Variable	n	mean	SD	n	mean	SD	n	mean	SD
Age category, years									
20–39	3	$4,876.16	$1,504.47	3	$3,850.82	$1,577.68	3	$1,025.34	$885.64
40–59	56	$6,885.60	$7,610.06	55	$3,332.71	$2,935.04	56	$3,602.92	$6,764.20
60–79	110	$6,809.96	$8,094.56	110	$2,600.37	$1,982.24	111	$4,220.13	$7,820.28
80+	15	$5,848.98	$3,761.21	16	$2,248.88	$1,793.78	15	$3,236.11	$3,918.42
Total	184	*KW 0*.*284*	*p = 0*.*963*	183	*KW 4*.*627*	*p = 0*.*201*	185	*KW 6*.*196*	*p = 0*.*102*
Sex									
Female	86	$5,367.16	$6,494.25	86	$2,638.77	$1,810.63	87	$2,758.87	$6,197.72
Male	100	$7,923.98	$8,352.11	99	$3,031.27	$2,811.92	100	$4,917.71	$7,798.53
Total	186	***MWU 3546***	***p = 0*.*039***	185	*MWU 4157*	*p = 0*.*783*	187	*MWU 3655*	*p = 0*.*060*
Cancer type									
Breast	32	$5,994.02	$7,423.98	32	$2,660.86	$1,757.45	32	$3,333.17	$7,113.69
Lung	27	$7,762.61	$9,037.45	27	$2,949.56	$1,730.09	27	$4,813.05	$8,779.30
Head and neck	15	$7,424.40	$7,825.39	14	$5,944.05	$4,595.07	15	$1,841.22	$4,321.68
Other	30	$7,171.41	$8,187.57	30	$3,149.96	$2,578.33	30	$4,021.45	$8,098.20
Gastrointestinal	29	$4,836.91	$3,506.13	29	$2,609.51	$1,865.06	29	$2,227.40	$3,061.78
Genotiurinary	41	$6,648.00	$6,775.18	41	$2,115.92	$1,852.36	41	$4,532.08	$6,096.31
Haematologic	7	$5,573.17	$8,563.87	7	$2,090.40	$951.23	7	$3,482.77	$8,588.70
Total	181	*KW 3*.*581*	*P = 0*.*733*	180	***KW 15*.*007***	***P = 0*.*020***	181	***KW 14*.*321***	***P = 0*.*026***
Pain NRS									
Moderate (2–4)	63	$6,670.87	$7,464.38	63	$2,938.57	$2,896.84	63	$3,732.30	$7,202.50
Severe (5–10)	123	$6,778.12	$7,755.65	122	$2,802.47	$2,111.71	124	$4,005.30	$7,169.09
Total	186	*MWU 3825*	*p = 0*.*887*	185	*MWU 3543*	*p = 0*.*385*	187	*MWU 3667*	*p = 0*.*494*

KW = Kruskal-Wallis; MBS = Medicare Benefits Schedule; MWU = Mann-Whitney U; NRS = numeric rating scale; PBS = Pharmaceutical Benefits Schedule; SD = standard deviation. Shaded cells indicate statistically significant differences.

Mean total MBS and PBS costs varied by cancer type. Mean total MBS costs were highest for participants diagnosed with head and neck cancers ($5,944, 95% CI $3,291, $8,597), whereas mean total PBS costs were highest for people diagnosed with lung cancers ($4,813, 95% CI $1,340, $8,286). However, there were no other differences detected between PBS and MBS costs for age, gender or baseline pain level.

#### 3. Relationship between healthcare costs and pain intensity

Pain intensity was not associated with healthcare costs after adjusting for age and sex (see Table 6 in S4 Appendix). However, after controlling for age, mean total healthcare costs were $2,668 higher for men than women (95% CI $461, $4,875, p = 0.02; Model 1, see Table 6 in S4 Appendix). Men also had higher mean total healthcare costs than women when adjusting for age, cancer type and baseline pain levels in the exploratory analyses (p<0.01; Model 2). No other variables were associated with healthcare costs after adjusting for age and sex.

Pain intensity was also not associated with mean total MBS costs after adjusting for age, sex and cancer type (see Table 7 in S5 Appendix). However, after controlling for age, gender and pain intensity, mean total MBS costs were associated with cancer type (p = 0.03) (see Table 7 in S5 Appendix). Men had higher mean total PBS costs than women when adjusting for age, cancer type and baseline pain levels (p<0.01). No other variables were associated with MBS or PBS costs after adjusting for the other co-variates.

Exploratory, *post hoc* adjusted analyses of the MBS and PBS costs by category using two-part models [70, 71] to account for the substantial proportion of zero-cost observations suggested only therapeutic procedures (including radiation oncology and therapeutic nuclear medicine; p = 0.05) and antineoplastic and immunomodulating agent costs (p = 0.04) were higher for men than women. Diagnostic imaging service costs were lowest for haematological cancers (p<0.01). No other MBS of PBS category costs differed by cancer type.

## Discussion

The findings suggest government funded, out-of-hospital costs are, on average, $2,247 per month for people living with advanced cancer and pain, i.e. approximately $27,000 per year, higher than recently reported MBS and PBS costs for the first 12-months following cancer diagnoses in Queensland, Australia (approximately 2012 A$7,224 per person per year) [55]. Advances in cancer care such as new immunotherapy drugs and increasing prices for new cancer drugs which have more than doubled in the past decade [72], may account for some of these differences and variations in the coverage of cancer services between New South Wales and Queensland [73]. Of note, outside of opioids, the proportion of excess costs due to pain cannot be separated from cancer care costs. Medications accounted for a slightly higher proportion of the costs [58%] relative to medical and allied health professional services and investigations (42%). Medications were also the most frequently utilised healthcare resource, on average 5.7 prescriptions per participant per month, followed by pathology services (average 5.6) and professional attendances (average 3.0).

A smaller proportion of the Stop cancer PAIN Trial participants received concessional benefits compared with the Australian general population, suggesting patients with advanced cancer could incur greater out-of-pocket expenses [22]. Cancer has been shown to cause substantial financial burden to individuals across many countries with diversely funded health systems [21, 24] and are a particular problem for people with advanced disease [74]. In addition to associated healthcare costs, pain has been shown to have financial impacts through reduced employment, at least in the cancer survivor context [75]. More research is needed to quantify the financial implications and impact on wellbeing for Australian patients living with advanced cancer, with and without pain, to help inform the development of appropriate policies, programs and strategies for improving financial wellbeing in this population.

Three of the ten most commonly prescribed medications in the sample were the same as those for the general Australian population in 2019–20; pantoprazole and esomeprazole which are largely prescribed for peptic ulcers and gastroesophageal reflux disease, and rosuvastatin for lowering high cholesterol levels [76]. This prescribing pattern is consistent with previous evidence suggesting potentially clinically futile treatments in people with advanced cancer include gastric protectors and statins [77–79]. Proton pump inhibitors (PPI) like pantoprazole can alter the gut microbiome and may decrease the efficacy of some oral cancer treatments [80]. Consequently, further investigation is warranted given almost half of the sample (48.9%) were treated with a PPI. The PBS data do not include medications purchased over the counter rather than via prescription such as non-steroidal anti-inflammatory drugs for pain which can increase reflux and gastric ulceration and PPI use [81]. Further, PPIs can been prescribed to reduce the adverse effects of corticosteroids which were the fourth most commonly supplied medications (Fig 1) [82–85].

More than one in five people with advanced cancer and pain were prescribed a lipid modifying agent, contrary to guidance to reduce the burden of medications in advanced disease, particularly from medications such as statins which are only prescribed for long term population-level risk reduction [86–89]. Further, discontinuing statins may improve quality of life [90] and people may be more likely to continue the medications that they most need. Targeted strategies are required to support the deprescribing of potential futile treatments such as implementing a deprescribing tool acceptable in clinical practice [91, 92].

The remaining top ten most frequently prescribed medications in the study sample were related to the treatment of pain (or, to a lesser extent, breathlessness [93, 94]; 50%), nausea and vomiting (10%) and constipation (10%). Dexamethasone, a commonly used corticosteroid which can be prescribed for multiple indications such as palliation of symptoms due to raised intracranial pressure, premedication before chemotherapy and antiemesis after chemotherapy and anorexia and nausea [95–97], was the seventh most commonly prescribed medication. Regretfully, the PBS data do not include information on the reasons for prescribing the medication. A recent study from New Zealand suggested corticosteroids are largely prescribed based on anecdotal and experiential evidence rather than on robust research [98]. Further investigation into the specific reasons for prescribing dexamethasone in the Australian setting using audit or qualitative methods is warranted given potential harms, absence of evidence for prolonged use of dexamethasone and limited information on why prescribers choose this medication [97, 98].

Few factors were associated with total healthcare costs, contrary to findings reported in the US [11, 27]. Whilst pain intensity and age were associated with total healthcare costs previously, gender was the only baseline clinico-demographic variable related to total healthcare costs in this analysis. Differences in how healthcare is funded between the US and Australia and changes in treatment patterns over time may account for the divergent findings. Additionally, there may have been insufficient numbers of participants with lower pain scores to detect a relationship between pain intensity and healthcare costs. Further exploration into the relationship between changes in pain intensity and healthcare costs is needed to more accurately predict how better management of pain in people with advanced cancer may impact healthcare resource utilisation.

Differences in total healthcare costs between males and females with advanced cancer and pain is consistent with previous evidence which suggests a gender difference in total healthcare resource utilisation and costs for people living with cancer [99, 100]. Previous studies suggest women are less likely to have surgery at the time of diagnosis and chemotherapy is used less often [99–102], consistent with the exploratory, *post hoc* findings. There is also limited evidence to suggest men and women perceive and respond to pain differently driven by biological, psychological and cultural factors [103, 104]. This observation warrants further investigation into sex differences in pain management, particularly given similar baseline pain scores, and exploration of the underpinning rationale for divergent findings to promote equitable access to cancer care. At present, sex is usually not taken into account in clinical decision making in oncology despite accumulating evidence that the individual’s sex is one of the most important factors influencing cancer risk and response to treatment [105].

Finally, patterns in MBS costs by cancer type are consistent with population-based estimates of health services costs for people receiving cancer care (with or without pain) in Australia which suggest healthcare costs vary by cancer type and time since diagnosis, possibly driven by differences in treatment modalities and frequency, new targeted therapies and immunotherapies, and associated tests and administrative MBS items [15].

### Strengths and limitations

Consent to access MBS and PBS data was granted by just under a third of study participants and findings may not reflect treatment patterns in the entire study cohort. However, unlike healthcare resource utilisation data collected using other means such as surveys, Medicare and PBS data are not prone to recall bias and typically provide greater accuracy than other methods of measuring costs [21, 106, 107]. Medicare data consent rates vary considerably [108] and may have been influenced by the level of study burden due to the number of study components and multiple consent forms required to access the data [9]. All analysis were conducted on available data only, i.e., methods for missing data were not applied. As there was no statistical or clinical difference in pain-related outcomes between the intervention and control groups in the Stop Cancer PAIN trial, participants were pooled for this analysis. Further, the quality of life data should be interpreted with caution as there was a sizable proportion of missing data for the C15-PAL, where physically unwell patients may be less likely to respond [109]. Cost data are reported for the three months prior to screening commensurate with previous cost analyses to account for sufficient variation in resource use when estimating the average cost per month [11, 57–59] and pain scores at screening may not accurately reflect the average pain intensity experienced during this time period. The MBS and PBS data do not include the costs of cancer services provided by the state or non-government agencies which may underestimate the costs of chemotherapy or costs borne by the patient and informal carers such as over the counter medications and lost income. Further, the cost of emergency department presentations and hospital admissions are not included in MBS and PBS data, nor medications not covered under the PBS. The findings therefore underestimate the total economic burden associated with advanced cancer pain. Further research is needed to elucidate a more complete picture of the healthcare services and costs associated with the management of pain in people with advanced cancer. Finally, generalisability of the results will be limited to similar healthcare and costing and funding models and similar populations. For example, a smaller proportion of participants received concessional payments compared with the general public, possibly reflecting a more affluent study population, particularly given evidence of inequalities in access to clinical trials [110] and cancer care more broadly in Australia [111, 112].

Despite these caveats, this analysis provides valuable insights into government funded out-of-hospital costs associated with advanced cancer pain to inform priority setting and policy development.

### Implications for research and practice

The findings identify areas of treatment for outpatients with advanced cancer and pain requiring further exploration and practice change, particularly the high use of peptic ulcer/GORD drugs, lipid modifying agents and corticosteroids. Further research is needed to determine why healthcare costs were higher in men than women with advanced cancer and experiencing pain and to explore both sex and gender-based differences and provider related factors.

Health economic research which includes costs related to emergency department and hospital admissions is needed. The authors recommend that future research evaluating interventions to improve pain outcomes include a health economic analysis. Also, the cost effectiveness of many evidence-based non-pharmacological interventions needs further research.

## Conclusions

This study provides vital information for informing quality of care and quality use of medicines, resource allocation and developing sustainable health policy. There was no clear relationship between pain intensity and healthcare costs demonstrated in this population with pain. Investigation into the underpinning rationale for higher healthcare costs in men is needed to promote equitable access to cancer care.

## Supporting information

S1 Appendix Table 1 medicare benefits schedule categories. (DOCX)

S2 Appendix Fig 1 proportion of the study sample who utilised government funded medical services as classified by the 2020 medicare benefits schedule. (DOCX)

S3 Appendix Fig 2 proportion of the study sample who were supplied with government-subsidised medicines categorised according to the World Health Organisation Anatomical Therapeutic Chemical (ATC) classification system. (DOCX)

S4 Appendix Table 6 association between clinico-demographics and mean total healthcare costs. (DOCX)

S5 Appendix Table 7 association between clinico-demographics and mean total MBS and PBS costs. (DOCX)
