# Supplementary material for: Treatment patterns and out-of-hospital healthcare resource utilisation by patients with advanced cancer living with pain: An analysis from the Stop Cancer PAIN trial
Source: PLoS One. 2023 Feb 28;18(2):e0282465. doi: 10.1371/journal.pone.0282465 (PMC9974128; doi:10.1371/journal.pone.0282465)
Supplement: S1 Appendix — (DOCX) [file pone.0282465.s001.docx]

**S1 Appendix Table 1 Medicare Benefits Schedule Categories (July 2019) (25)**

| **Category** | **Group** | **Description** |  |
| --- | --- | --- | --- |
| **1** | **Professional attendances** | | |
|  | A1 | General practitioner attendances to which no other item applies |  |
|  | A2 | Other non-referred attendances to which no other item applies |  |
|  | A3 | Specialist attendances to which no other item applies |  |
|  | A4 | Consultant physician attendances to which no other item applies |  |
|  | A5 | Prolonged attendances to which no other item applies |  |
|  | A6 | Group therapy |  |
|  | A7 | Acupuncture & non-specialist practitioner items |  |
|  | A8 | Consultant psychiatrist attendances to which no other item plies |  |
|  | A9 | Contact lenses – attendances |  |
|  | A10 | Optometrical services |  |
|  | A11 | Urgent attendance after hours |  |
|  | A12 | Consultant occupational physician attendances to which no other item applies |  |
|  | A13 | Public health physician attendances to which no other item applies |  |
|  | A14 | Health assessments |  |
|  | A15 | GP management plans, team care arrangements, multidisciplinary care plans |  |
|  | A17 | Domiciliary & residential management reviews |  |
|  | A18 | General practitioner attendance associated with pip incentive payments |  |
|  | A19 | Other non-referred attendances associated with pip incentive payments to which no other item applies |  |
|  | A20 | GP mental health treatment |  |
|  | A21 | Medical practitioner (emergency physician) attendances to which no other item applies |  |
|  | A22 | General practitioner after-hours attendances to which no other item applies |  |
|  | A23 | Other non-referred after-hours attendances to which no other item applies |  |
|  | A24 | Pain & palliative medicine |  |
|  | A26 | Neurosurgery attendances to which no other item applies |  |
|  | A27 | Pregnancy support counselling |  |
|  | A28 | Geriatric medicine |  |
|  | A29 | Early intervention services for children with autism, pervasive developmental disorder or disability |  |
|  | A30 | Medical practitioner (including a general practitioner, specialist or consultant physician) telehealth attendances |  |
|  | A31 | Addiction medicine |  |
|  | A32 | Sexual health medicine |  |
|  | A33 | Transcatheter aortic valve implantation case conference |  |
|  | A34 | Health care homes |  |
|  | A35 | Services for patients in residential aged care facilities |  |
| **2** | **Diagnostic procedures** | | |
|  | D1 | Miscellaneous diagnostic procedures and investigations |  |
|  | D2 | Nuclear medicine (non-imaging) |  |
| **3** | **Therapeutic procedures** | | |
|  | T1 | Miscellaneous therapeutic procedures |  |
|  | T2 | Radiation Oncology |  |
|  | T3 | Therapeutic nuclear medicine |  |
|  | T4 | Obstetrics |  |
|  | T6 | Anaesthetics |  |
|  | T7 | Regional or field nerve blocks |  |
|  | T8 | Surgical operations |  |
|  | T9 | Assistance at operations |  |
|  | T10 | Relative value guide for anaesthesia |  |
|  | T11 | Botulinum toxin injections |  |
| **4** | **Oral & maxillofacial services** | | |
|  | O1 | Consultations |  |
|  | O2 | Assistance at operation |  |
|  | O3 | General surgery |  |
|  | O4 | Plastic & reconstructive |  |
|  | O5 | Preprosthetic |  |
|  | O6 | Neurosurgical |  |
|  | O7 | Ear, nose & throat |  |
|  | O8 | Temporomandibular joint |  |
|  | O9 | Treatment of fractures |  |
|  | O11 | Regional or field nerve blocks |  |
| **5** | **Diagnostic imaging services** | | |
|  | I1 | Ultrasound |  |
|  | I2 | Computed tomography |  |
|  | I3 | Diagnostic radiology |  |
|  | I4 | Nuclear medical imaging |  |
|  | I5 | Magnetic resonance imaging |  |
|  | I6 | Management of bulk-billed services |  |
| **6** | **Pathology services** | | |
|  | P1 | Haematology |  |
|  | P2 | Chemical |  |
|  | P3 | Microbiology |  |
|  | P4 | Immunology |  |
|  | P5 | Tissue pathology |  |
|  | P6 | Cytology |  |
|  | P7 | Genetics |  |
|  | P8 | Infertility & pregnancy tests |  |
|  | P9 | Simple basic pathology tests |  |
|  | P10 | Patient episode initiation |  |
|  | P11 | Specimen referred |  |
|  | P12 | Management of bulk-billed service |  |
|  | P13 | Bulk-billing incentive |  |
| **7** | **Cleft lip & cleft palate services** | | |
|  | C1 | Orthodontic services |  |
|  | C2 | Oral & maxillofacial services |  |
|  | C3 | General & prosthodontic services |  |
| **8** | **Miscellaneous services** | | |
|  | M1 | Management of bulk-billed services |  |
|  | M3 | Allied health services |  |
|  | M6 | Psychological therapy services |  |
|  | M7 | Focused psychological strategies (allied mental health) |  |
|  | M8 | Pregnancy support counselling |  |
|  | M9 | Allied health group services |  |
|  | M10 | Autism, pervasive developmental disorder & disability services |  |
|  | M11 | Allied health services for indigenous Australians who have had a health check |  |
|  | M12 | Services provided by a practice nurse or Aboriginal & Torres Strait Islander Health Practitioner on behalf of a medical practitioner |  |
|  | M13 | Midwifery services |  |
|  | M14 | Nurse practitioners |  |
|  | M15 | Diagnostic audiology services |  |
