# Supplementary material for: Treatment patterns and out-of-hospital healthcare resource utilisation by patients with advanced cancer living with pain: An analysis from the Stop Cancer PAIN trial
Source: PLoS One. 2023 Feb 28;18(2):e0282465. doi: 10.1371/journal.pone.0282465 (PMC9974128; doi:10.1371/journal.pone.0282465)
Supplement: S2 Appendix — (DOCX) [file pone.0282465.s002.docx]

**S2 Appendix Figure 1 Proportion of the study sample who utilised government funded medical services as classified by the 2020 Medicare Benefits Schedule** (note, there were no services reported for “7. Cleft lip & cleft palate services”)
