# Supplementary material for: Treatment patterns and out-of-hospital healthcare resource utilisation by patients with advanced cancer living with pain: An analysis from the Stop Cancer PAIN trial
Source: PLoS One. 2023 Feb 28;18(2):e0282465. doi: 10.1371/journal.pone.0282465 (PMC9974128; doi:10.1371/journal.pone.0282465)
Supplement: S3 Appendix — (DOCX) [file pone.0282465.s003.docx]

**S3 Appendix Figure 2 Proportion of the study sample who were supplied with government-subsidised medicines categorised according to the World Health Organisation Anatomical Therapeutic Chemical (ATC) classification system**
