# Supplementary material for: Treatment patterns and out-of-hospital healthcare resource utilisation by patients with advanced cancer living with pain: An analysis from the Stop Cancer PAIN trial
Source: PLoS One. 2023 Feb 28;18(2):e0282465. doi: 10.1371/journal.pone.0282465 (PMC9974128; doi:10.1371/journal.pone.0282465)
Supplement: S4 Appendix — (DOCX) [file pone.0282465.s004.docx]

**S4 Appendix Table 6 Association between clinico-demographics and mean total healthcare costs**

|  | **Adjusted for age and sex (Model 1)** | | | | **Full model (Model 2)** | | |
| --- | --- | --- | --- | --- | --- | --- | --- |
| **Variables** | EMM | 95% CI | p-value | EMM | | 95% CI | p-value |
| Age |  |  | 0.358 |  | |  | 0.063 |
| Sex |  |  | **0.019** |  | |  | **<0.001** |
| Female | $5,275.57 | $4,062.00, $6,851.71 |  | $4,492.56 | | $3,716.49, $5,430.71 |  |
| Male | $7,943.51 | $6,435.10, $9,805.52 |  | $7,871.47 | | $6,150.64, $10,073.75 |  |
| Cancer type |  |  |  |  | |  | 0.191 |
| Breast |  |  |  | $7,535.98 | | $4,649.75, $12,213.77 |  |
| Lung |  |  |  | $7,890.51 | | $5,415.49, $11,496.66 |  |
| Head & neck |  |  |  | $5,396.66 | | $3,383.83, $8,606.81 |  |
| Other |  |  |  | $6,362.77 | | $4,649.51, $8,707.34 |  |
| Gastrointestinal |  |  |  | $4,562.42 | | $3,566.02, $5,837.24 |  |
| Genitourinary |  |  |  | $6,165.90 | | $4,464.44, $8,515.82 |  |
| Haematological |  |  |  | $4,578.43 | | $2,106.45, $9,951.35 |  |
| Pain NRS |  |  |  |  | |  | 0.296 |
| Moderate |  |  |  | $6,433.80 | | $5,031.92, $8,226.25 |  |
| Severe |  |  |  | $5,496.45 | | $4,511.62, $6,696.25 |  |
| Model fit |  |  |  |  | |  |  |
| Constant | 9.39 |  |  | 9.42 | |  |  |
| AIC | 3606.63 |  |  | 3496.40 | |  |  |
| BIC | 3619.49 |  |  | 3531.46 | |  |  |
| LL | -1799.32 |  |  | -1737.20 | |  |  |
| Likelihood ratio | 8.70 | p=0.013 |  | 20.78 | | p=0.014 |  |

AIC = Akaike information criteria; BIC = Bayesian information criteria; EMM = estimated marginal means; LL = log likelihood; NRS = numeric rating scale; covariates in the model are fixed at age=64.32
