# Supplementary material for: Treatment patterns and out-of-hospital healthcare resource utilisation by patients with advanced cancer living with pain: An analysis from the Stop Cancer PAIN trial
Source: PLoS One. 2023 Feb 28;18(2):e0282465. doi: 10.1371/journal.pone.0282465 (PMC9974128; doi:10.1371/journal.pone.0282465)
Supplement: S5 Appendix — (DOCX) [file pone.0282465.s005.docx]

**S5 Appendix Table 7 Association between clinico-demographics and mean total MBS and PBS costs**

|  | **MBS** | | | **PBS** | | |
| --- | --- | --- | --- | --- | --- | --- |
| **Variables** | EMM | 95% CI | p-value | EMM | 95% CI | p-value |
| Age |  |  | 0.202 |  |  | 0.113 |
| Sex |  |  | 0.133 |  |  | **0.002** |
| Female | $2,583.51 | $2,230.61, $2,992.24 |  | $2,033.78 | $1,204.78, $2,862.80 |  |
| Male | $3,040.93 | $2,577.19, $3,588.11 |  | $5,549.44 | $3,125.25, $7,973.64 |  |
| Cancer type |  |  | **0.026** |  |  | 0.057 |
| Breast | $2,603.13 | $2,014,57, $3,363.64 |  | $5,399.72 | $1,074.52, $9,724.92 |  |
| Lung | $2,948.58 | $2,330.80, $3,8730.10 |  | $5,388.75 | $1,527.37, $9,250.13 |  |
| Head & neck | $4,798.04 | $3,314.73. $7,343.92 |  | $881.27 | -$11.66, $1,774.20 |  |
| Other | $3,026.44 | $2,360.13, $3,880.87 |  | $3,623.60 | $1,160.28, $6,086.92 |  |
| Gastrointestinal | $2,733.34 | $2,068.17. $3,612.45 |  | $2,456.55 | $788.76, $4,124.35 |  |
| Genitourinary | $2,158.35 | $1,664.42, $2,798.86 |  | $4,348.82 | $1,788.30, $6,909.34 |  |
| Haematological | $2,066.98 | $1,514.30, $2,821.39 |  | $2,262.32 | -$802.53, $5,327.17 |  |
| Pain NRS |  |  | 0.621 |  |  | 0.737 |
| Moderate | $2,890.35 | $2,370.89, $3,523.62 |  | $3,971.29 | $1,940.11, $6,002.47 |  |
| Severe | $2,718.11 | $2,388.13, $3,093.67 |  | $3,566.23 | $2,253.70, $4,878.77 |  |
| Model fit |  |  |  |  |  |  |
| Constant | 8.07 |  |  | 9.53 |  |  |
| AIC | 3568.39 |  |  | 3256.69 |  |  |
| BIC | 3604.73 |  |  | 3288.56 |  |  |
| LL | -1773.20 |  |  | -1618.34 |  |  |
| Likelihood ratio | 21.21 | p=0.012 |  |  |  |  |

AIC = Akaike information criteria; BIC = Bayesian information criteria; EMM = estimated marginal means; LL = log likelihood; MBS = Medicare Benefits Schedule; NRS = numeric rating scale; PBS = Pharmaceutical Benefits Schedule; covariates in the model are fixed at age=64.32
